# Aflatoxin M1 Content and Mastitis-Causing Bacteria in Milk from Skopelos Dairy Goats Reared in Extensive and Intensive Farming Systems

**DOI:** 10.3390/ani15091238

**Published:** 2025-04-28

**Authors:** Ioannis Stavropoulos, Zoitsa Basdagianni, Georgios Manessis, Aikaterini Tsiftsi, Ioannis Bossis

**Affiliations:** Laboratory of Animal Husbandry, Department of Animal Production, School of Agriculture, Aristotle University of Thessaloniki, 54124 Thessaloniki, Greece; basdagianni@agro.auth.gr (Z.B.); gmanes@agro.auth.gr (G.M.); atsifts@agro.auth.gr (A.T.); bossisi@agro.auth.gr (I.B.)

**Keywords:** goat milk, aflatoxin M1, farming system, milk quality, mastitis microorganisms, Skopelos breed

## Abstract

This study assessed the impact of farming systems on aflatoxin M1 (AFM1) content and the prevalence of mastitis-causing bacteria in goat milk. A total of 233 milk samples were collected from two Skopelos goat farms—one intensive and one extensive farm—and analyzed for AFM1 content using a competitive ELISA. An additional 219 samples from goats suspected of subclinical mastitis were tested for bacterial prevalence with microbial culturing. The results showed that AFM1 concentration was significantly higher in the intensive farming system (7.76 ± 0.76 ng/kg) than in the extensive farming system (3.78 ± 0.79 ng/kg), though it remained below the legal limit of 50 ng/kg. The main effects of season and year were not significant, though higher levels of AFM1 were observed during winter. The interaction effects of season–farming system and year–season–farming system on AFM1 levels were significant. The prevalence of mastitis-causing bacteria varied by system, with *Streptococcus* spp. being more common in the extensive farming system, and *Staphylococcus aureus* was more frequently detected in milk samples from the intensive farming system. Binomial regression indicated that both the farming system and lactation stage significantly influenced *Streptococcus* spp. prevalence (*p* = 0.05; OR = 1.9 and 2.7, respectively). It is concluded that the farming system affects those quality parameters in goat milk.

## Simple Summary

Mycotoxins are toxic substances produced by molds on feed. Mycotoxins ingested by dairy animals are transformed into aflatoxin M1 (AFM1) and pass onto milk, degrading its quality and safety. Pathogens such as Streptococci and Staphylococci contaminate milk through mastitis or poor milking practices. Understanding the factors influencing the presence of mycotoxins and pathogens in milk is crucial for improving dairy quality and ensuring consumer safety. This study investigated the effects of the farming system (intensive vs. extensive), year, and season on AFM1 levels as well as the impact of the farming system on the prevalence of mastitis-causing pathogens. The results revealed that AFM1 content was significantly higher in the intensive farming system, potentially due to feeding and storage practices. Interactions between year and season had a notable impact on AFM1 levels, which may reflect variations in climate conditions and animal management strategies over time. Milk AFM1 levels in both farming systems remained below legal thresholds. The prevalence of bacterial pathogens was significantly influenced by both the farming system and the stage of lactation. *Streptococcus* spp. appeared more frequently in the extensive system, potentially due to hand-milking practices. Its presence increased as lactation progressed, possibly due to heightened udder susceptibility in the later stages.

## 1. Introduction

Milk and its derivatives are viewed as staple foods in human diets due to their high nutritional value. In this context, milk safety is paramount due to its susceptibility to spoilage and contamination, including mycotoxins and pathogenic strains of bacteria. Mycotoxins are toxic secondary metabolites produced by molds such as *Aspergillus* spp. and pose a significant animal and human health hazard due to their potential carcinogenic properties and hepatotoxicity [1,2,3,4,5]. Among mycotoxins, aflatoxin B1 (AFB1) is a primary concern, given that it undergoes metabolic conversion to aflatoxin M1 (AFM1) in the animal’s liver, which subsequently passes to milk [3,6]. Cereal grains and forages, commonly used in ration formulation, serve as ideal substrates for mold growth, leading to mycotoxin ingestion by dairy animals [7]. Furthermore, in intensive or semi-intensive production systems, meeting the protein and energy requirements is not possible exclusively through forage consumption [8,9]. This results in purchasing and storing for long periods of time large quantities of concentrates and roughages, thus increasing aflatoxin-associated risks [5]. Moreover, AFB1 and AFM1 levels in feedstuffs and milk, respectively, demonstrate climate and seasonal variations [10,11].

Many studies on aflatoxin M1 highlight its resistance to thermal processing [12,13,14], indicating that standard industry processes such as pasteurization are insufficient to mitigate this risk. Moreover, aflatoxins bind to casein micelles and remain intact during cheese making, even when pasteurized milk is used in the process [15,16,17]. For example, in Feta cheese, aflatoxins are slightly reduced after pasteurization and brining but can still be traced at elevated levels in the curd [18]. In this context, food safety and health authorities worldwide have set limits for aflatoxins in food. However, in milk, limits have been set only for AFM1. The European Food Safety Authority (EFSA) allows up to 50 ng/kg of aflatoxin M1 in raw and pasteurized milk (EC No 1881/2006) [19]. A stricter limit of 25 ng/kg has been set for infant milk. In comparison, the Food and Drug Administration (FDA) has set the limit for aflatoxin M1 in milk at 500 ng/kg [20].

Another important issue for public health associated with the dairy goat industry is the consumption of raw milk or cheese products originating from unpasteurized milk, which might be contaminated by pathogenic strains of bacteria [21]. Those pathogenic bacteria can be of environmental origin or part of the gland’s microflora. This often results in udder inflammation (mastitis) and consequently in milk and dairy product degradation [8,9,22,23,24]. For this reason, the dairy industry and legislative authorities have defined limits for relevant indicators such as the Total Bacterial Count (TBC) and Somatic Cell Count (SCC), which are related to the presence of bacteria and mastitis, respectively [10].

Regulations also foresee other limits related to bacteria in milk. For example, dairy products should be free of Staphylococci toxins or have limited counts of *E. coli* and other bacteria belonging to the *Enterobacteriaceae* family (EC No 2073/2005) [11]. *Staphylococcus aureus*, which is highly pathogenic and resistant to several antibiotics, has also been the center of attention of food and drug authorities [21,25,26,27,28]. The thermal processing (pasteurization) of milk may be lethal for the microorganism but fails to inactivate excreted toxins [29,30,31]. Other important milk bacteria, which are related to mastitis, are species of *Streptococcus*, non-hemolytic strains of *Staphylococcus*, and some coliform species [32]. Although these microorganisms are normally found on the udder exterior, they also have been associated with mastitis or subclinical cases [33,34,35]. It is also hypothesized that various milking practices may facilitate the entry of bacterial strains into the udder through contaminated equipment or the farmers’ hands, potentially leading to inflammation.

While numerous studies have explored aflatoxins and bacteria in bovine milk, research on dairy goats—particularly regarding the impact of farming systems (intensive vs. extensive) and seasonality—remains limited. This study aspires to provide relevant insights by examining the effects of farming system, season, and year on AFM1 levels in milk and the presence of mastitis-causing bacteria (Gram-positive hemolytic cocci—*Staphylococcus* spp., *Streptococcus* spp., enterobacteria—*E. coli*, and non-hemolytic *Staphylococcus* (*CNS*)) in two Skopelos goat farms managed under extensive and intensive systems.

## 2. Materials and Methods

### 2.1. Sampling and Sample Processing

The study was conducted on two Greek goat farms with different farming systems. The first farm, located in the Attica region (23.95° E, 37.95° N), followed an intensive system where goats had no access to outdoor places and were fed commercial concentrates, alfalfa hay, and wheat straw. The second farm, situated on Skopelos island (23.7° E, 39.14° N), operated under an extensive system, allowing goats to graze in grasslands and shrublands throughout the year. Machine milking was applied on the intensive farm, while on the extensive one, milking was performed by hand.

A total of 60 adult Skopelos goats, a Greek native goat breed, in 2nd and 3rd lactation with close parturition date, were randomly selected for aflatoxin analysis (AFM1). Milk samples were collected two times per lactation, after weaning in February (winter) and during mid-lactation in June (early summer), across two consecutive years (2022 and 2023). Four sampling occasions were scheduled for the two-year study and 233 milk samples were collected (112 milk samples from extensive and 121 from the intensive farming system). After collection, milk samples were stored at −20 °C awaiting analyses for aflatoxin content.

In addition to AFM1 sampling, further milk samples were collected to assess the existence of specific mastitis-causing bacteria. For this purpose, 100 goats per farming system were randomly selected, and milk sampling was conducted at four lactation stages—after weaning, peak lactation, mid-lactation, and late lactation—for somatic cell count (SCC) analysis. Goats with SCC levels exceeding 1.5 × 10^6^ cells/mL in the previous sampling were considered at risk for clinical or subclinical mastitis, and their milk samples were subjected to further bacteriological analysis to identify infectious and/or environmental pathogens in milk. Over the two-year study period, six sampling occasions were scheduled for this analysis, resulting in the collection of 219 milk samples (148 from the extensive system and 71 from the intensive system).

### 2.2. Analysis of Aflatoxin Content

The quantification of aflatoxin M1 (AFM1) was performed using a commercial ELISA Kit (Aflatoxin M1 Detection ELISA Kit, 96 wells, 961AFLM01M-96, ASTORI S.n.c., Poncarale, Italy). The absorbance of standards and samples was measured with a microplate reader (Chromate 4300, Awareness Technology, Inc., Palm City, FL, USA using a primary filter (450 nm) and a differential filter (630 nm). The assay involved the use of 6 standards (0, 5, 10, 25, 50, 100 pg/mL of aflatoxin M1 in aqueous solution) to estimate the standard curve and the AFM1 concentration of samples (pg/mL). The concentration was then converted to ng/kg, as is often expressed in food industry regulations. Aflatoxin M1 (AFM1) levels in goat milk were categorized into four concentration ranges: <5 ng/kg, <25 ng/kg, 25–50 ng/kg, and >50 ng/kg.

### 2.3. Bacterial Cultures

SCC was measured using a Fossomatic 5000 analyzer (Foss Electric, Hillerød, Denmark). For the identification of infectious and/or environmental pathogens in milk, a culture was carried out by the “spread plate method”. This method involved the streaking of milk samples on agar plates (Linearcount 3M^®^, Zoetis Inc., Parsippany, NJ, USA) with a 10 μL sterile inoculating l to obtain isolated bacterial colonies. Plates consisted of three wells, allowing simultaneous bacterial culturing in 3 different selective nutrient agars. These included (i) Columbia blood CNA agar for the detection of Gram-positive hemolytic cocci (*Staphylococcus* spp., *Streptococcus* spp.), (ii) Mac Conkey agar for the isolation of enterobacteria (*E. coli*, *Klebsiella* spp.), and (iii) Mannitol salt agar for the selective isolation of *Staphylococcus aureus* (differentiation from other non-hemolytic Staphylococcus species; CNS). Plates were incubated (B8058 incubator, Termaks, Bergen, Norway) at 37 °C for 24 h and afterward were inspected for the presence and morphology of bacterial colonies. The results were interpreted according to the manufacturer’s kit instructions.

### 2.4. Statistical Analysis

The results were tested for normality using the Kolmogorov–Smirnov test. The effect of farming system, season, and year on AFM1 was evaluated using the following linear mixed model:

Y_ijkl_ = μ + FS_i_ + S_j_ + Y_k_ + (FS × S × Y)_ijk_ + A_l_ + e_ijkl_ (1)

where Y_ijkl_ denotes the dependent variable (AFM1); μ is the overall mean; FS_i_ is the fixed effect of the farming system (i = 1–2); S_j_ is the fixed effect of the season (j = 1–2); Y_k_ is the fixed effect of the year (k = 1–2); (FS × Y × S)_iJk_ denotes the interaction effects of farming system, season and year; A_l_ is the random effects of the animal; and e_ijk_ is the residual error associated with observation ijk.

Regarding bacterial cultures, the prevalence of each microorganism was calculated. Percentages were calculated by dividing the number of positive samples by the total number of samples [36]. Statistical analysis was carried out using the chi-square test to investigate the prevalence of differences between the two farming systems. To assess the effect of the farming system, a binomial regression model was used. The farming system (intensive, extensive) was the primary explanatory variable, and the lactation stage (peak, middle, and ending phase) was included as a covariate to improve model fit. The presence or absence of mastitis-causing bacteria was treated as the binary outcome variable (1 = positive, 0 = negative). The model was evaluated for goodness-of-fit with the Hosmer–Lemeshow test. The data were analyzed in SPSS v.27. Significance was set at *p*-value < 0.05.

## 3. Results and Discussion

Table 1 indicates the distribution of the AFM1 levels of the analyzed samples for both farming systems.

In the extensive system, AFM1 concentrations were relatively low, with 81 samples containing AFM1 levels below 5 ng/kg and 31 samples over 5 ng/kg but below 25 ng/kg. Samples exceeding the 25 ng/kg threshold were not detected. In contrast, the intensive system showed higher AFM1 contamination, with 62 samples below 5 ng/kg, 54 samples over 5 ng/kg but below 25 ng/kg, and five samples exceeding 25 ng/kg (three between 25 and 50 ng/kg and two above 50 ng/kg). AFM1 concentrations in all samples remained below the proposed EFSA limit of 50 ng/kg [19].

Table 2 presents the mean concentrations (±standard error) of AFM1 in goat milk across different farming systems, seasons, and study years. The results demonstrate a significant effect of the farming system on aflatoxin contamination in both milk and feed. AFM1 concentrations in goat milk were significantly higher in the intensive system (7.76 ± 0.76 ng/kg) compared to the extensive system (3.78 ± 0.79 ng/kg) (*p* < 0.01) in line with the results obtained in other studies [37,38]. These findings suggest that intensive farming practices, where goats were fed with silage and concentrated mixtures of cereal grains, may contribute to higher aflatoxin contamination. Our results are in agreement with Bingol et al. (2007) [39] who reported higher levels (ppb) of aflatoxin in concentrates than in forages and increased AFM1 in the milk (ppt). On the other hand, CJ de Matos et al. (2021) [40] observed higher levels of AFM1 in milk from goats fed with forage (27.23 ng/L) than those fed with concentrates (19.08 ng/L).

Seasonal variations in aflatoxin levels were also observed (Table 2), with higher AFM1 concentrations recorded in winter (6.42 ± 1.1 ng/kg) compared to summer (5.12 ± 1.1 ng/kg), although the difference was not statistically significant (*p* = 0.21). This result is in agreement with Fallah et al. (2016), who highlighted the significant influence of cold seasons on AFM1 content in the milk of different species [41]. Asi et al. (2012) also stated that concentrations of AFM1 rose during cold seasons in cow, sheep, and goat milk [42]. The increased aflatoxin levels during winter may be attributed to the greater reliance on stored feed, which is more susceptible to fungal contamination and subsequent aflatoxin production [43,44].

Intensive conditions require controlled feeding and therefore the usage of commercial feedstuff, which contains large amounts of cereals. These materials are often susceptible to fungal infestation and aflatoxin production as a result of inappropriate storage conditions. In addition to changes in the humidity and ambient temperature of the storage areas, climate conditions and precipitation can also influence mycotoxin concentration in the feed or grass. Consequently, these conditions of high-producing farms may favor higher exposure to aflatoxins and the transference of AFM1 into cow [45,46] and goat milk [37]. Moreover, milk from modern cow and goat farms with upgraded features close to metropolitan areas showed higher levels of AFM1 compared to traditional, remote farms [38]. The inappropriate storage of feed constituents was referred to as a major contributor to fungal contamination and growth.

Yearly variations in aflatoxin contamination showed slightly higher AFM1 levels in 2022 (6.48 ± 0.76 ng/kg) compared to 2023 (5.04 ± 0.76 ng/kg) (*p* = 0.2). Although these differences were not statistically significant, they indicate possible variations in feed quality, storage conditions, or environmental factors affecting fungal growth across the two years.

A significant interaction effect was observed for AFM1 (*p* = 0.004), suggesting that farming system, season, and year do not act independently but rather interact to influence aflatoxin contamination levels. This finding highlights the complexity of aflatoxin contamination, which may be influenced by multiple factors, including environmental conditions, feed storage practices, and seasonal variations in feed composition.

**Figure 1:**
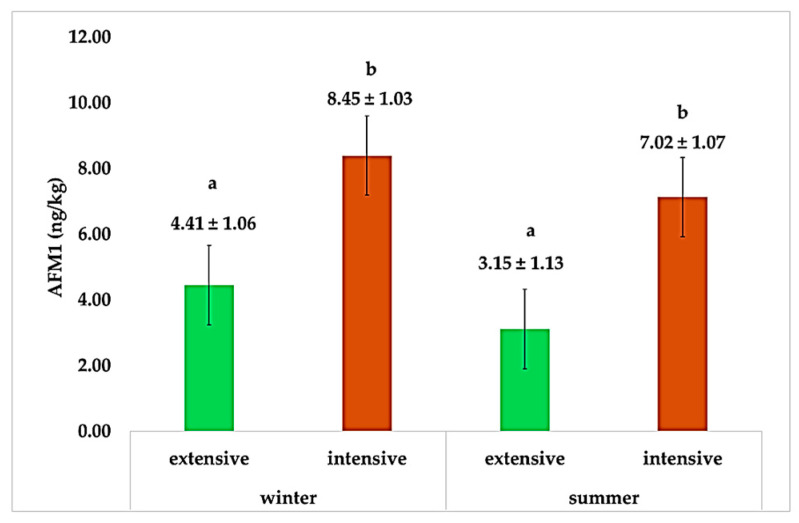
Mean content of AFM1 (ng/kg) in different seasons for each farming system. AFM1: aflatoxin M1 content in ng per kg ± standard Error; different letters above bars (a,b) indicate significant differences among groups.

Within seasons (Figure 1), our results indicate that in both winter and summer, AFM1 levels were higher in the intensive farming system compared to the extensive system. During winter, milk from the intensive system contained an average AFM1 concentration of 8.45 ± 1.03 ng/kg, which was significantly higher (*p* < 0.05) than the 4.41 ± 1.06 ng/kg observed in the extensive system. Similarly, in summer, AFM1 levels remained elevated in the intensive system (7.02 ± 1.07 ng/kg) compared to the extensive system (3.15 ± 1.13 ng/kg). The seasonal increase in AFM1 levels during winter can be attributed to the higher reliance on stored feed, which is more susceptible to fungal contamination compared to fresh forage availability in the summer and the very dry summer conditions of the region [47]. In winter, goats in the intensive system consume primarily commercial feedstuff, which, if improperly stored or contaminated, can result in higher mycotoxin levels in feed and consequently increased AFM1 levels in milk.

**Figure 2:**
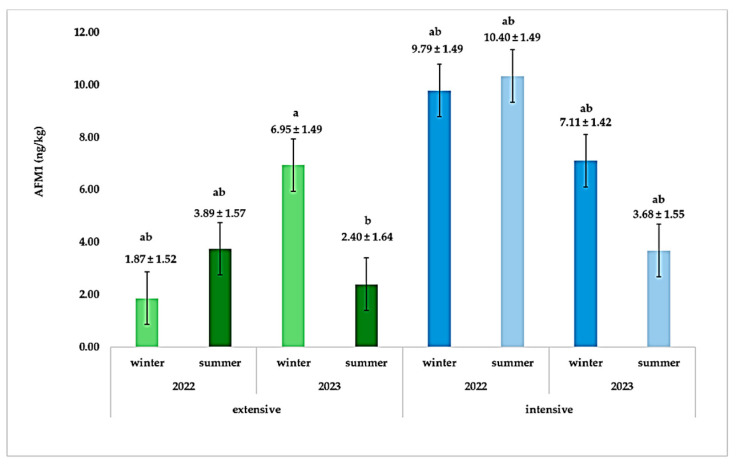
Average content of AFM1 (ng/kg) for each farming system and season between the two years. AFM1; aflatoxin M1 content in ng per kg ± standard Error; different letters above bars (a,b) indicate significant differences among groups.

In addition, the interaction between season, system, and year is significant, as AFM1 content in extensive and intensive systems varied significantly between two years for the same seasons (Figure 2). Specifically, in 2022, AFM1 levels in the extensive system were 1.87 ± 1.52 ng/kg in winter and increased to 3.89 ± 1.57 ng/kg in summer; however, the difference was not statistically significant (*p* > 0.05). In 2023, winter AFM1 levels in the extensive farming system rose to 6.95 ± 1.49 ng/kg, while summer levels dropped to 2.39 ± 1.64 ng/kg, a difference that was significant (*p* < 0.05). In the intensive system in 2022, AFM1 levels reached 9.8 ± 1.49 ng/kg in winter and rose to 10.4 ± 1.49 ng/kg in summer, but again, the difference was not statistically significant (*p* > 0.05). In 2023, the winter AFM1 levels remained elevated (7.11 ± 1.42 ng/kg), whereas summer levels decreased (3.68 ± 1.55 ng/kg), also without statistically significant differences.

These results confirm that winter poses a greater risk for AFM1 contamination in milk, likely due to the reliance on stored feed, which is more prone to fungal contamination [48]. The significantly higher AFM1 concentrations in the intensive system further reinforce the notion that commercial feed may have higher aflatoxin contamination compared to natural forage in the extensive system. Moreover, the year-to-year variation suggests that climatic conditions (differences in weather patterns) or feed storage practices may further influence aflatoxin contamination, as higher AFM1 levels were observed in winter 2023 compared to winter 2022 in the extensive system.

Regarding the identification of mastitis-causing bacteria, milk samples with somatic cell counts (SCC) exceeding 1.5 × 10^6^ cells/mL were selected for further analysis. This threshold was used as a criterion for identifying suspected cases of intramammary infection. A total of 148 milk samples were selected from animals in the extensive farming system suspected of having mastitis. Bacterial cultures confirmed that 46 samples tested positive exclusively for *Streptococcus* spp., 27 for *Staphylococcus aureus*, and 10 for non-hemolytic Staphylococci. Twenty-nine samples tested negative for bacterial infection. Mixed infections were also identified, including *S. aureus* with *Streptococcus* spp. (6 samples), *Streptococcus* spp. with non-hemolytic Staphylococci (28 samples), and *S. aureus* with *E. coli* (2 samples).

In the intensive farming system, 71 animals with suspected intramammary infection were examined. Culture results showed that 17, 21, and 5 samples were positive exclusively for *Streptococcus* spp., *S. aureus*, and non-hemolytic Staphylococci, respectively. Eighteen samples tested negative. Additionally, mixed infections were observed, including combinations of *S*. *aureus* with *Streptococcus* spp. (three samples) and *Streptococcus* spp. with non-hemolytic Staphylococci (seven samples). Those results were used to calculate the percentages of different bacterial infections.

**Figure 3:**
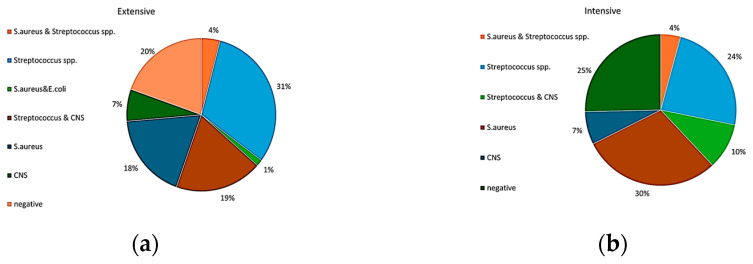
Percentages of different bacterial infections after identification in microbial cultures in (**a**) the extensive farm and (**b**) the intensive farm.

Therefore, samples positive only for *S. aureus* accounted for 30% and 18% in the intensive and extensive systems, respectively (Figure 3a,b). In the same order, samples positive for *Streptococcus* spp. accounted for 24% and 31% of the total samples analyzed. Lastly, positive samples only for CNS were found in 7% for both farming systems. In many samples, two different microorganisms were simultaneously detected. Most frequent co-infections included the presence of *Streptococcus* spp. and CNS, which accounted for 19% and 10% of positives in the extensive and intensive system, respectively. *Streptococcus* spp. and *S. aureus* isolates were identified together at a percentage of 4% in both systems. *E*. *coli* was found together with *S. aureus* only in the extensive farm (1%).

No statistically significant differences were found between the two farms for *S. aureus*, *E. coli*, and CNS using the chi-square test. However, there was a statistically significant difference (*p* < 0.05) for *Streptococcus* spp.

Table 3 presents the coefficients, standard errors, *p*-values, and odds ratios from a binomial regression model assessing the effect of the farming system on the presence of the aforementioned bacteria in goats suspected to have subclinical mastitis. The odds ratio revealed that goats of the extensive farming system with subclinical mastitis were more prone to Streptococcal infections (odds ratio = 1.9).

The Hosmer–Lemeshow test indicated a good fit for the model (*p* = 0.675). The results indicate that both the farming system and lactation stage influence the presence of *Streptococcus* spp. bacteria in goat milk samples.

In contrast to the findings of the current study, previous field studies reported a lower occurrence of *Streptococcus* spp. in goat milk [49,50]. Also, reports of bacteriological findings in the milk of extensive and low-input goat farms show a higher frequency of coagulase-positive Staphylococci and *E. coli* rather than *Streptococcus* spp. [32,50,51]. It is suggested that milking by hand poses a risk for mammary gland streptococcal infections in goats and the shedding of Streptococci in goat milk. This is a common practice in low-input traditional farms, as in the case of the extensive goat farm that was included in this study. *Streptococcus* species and *E. coli* have been reported by many authors as very capable microorganisms for udder inflammation that are usually present in mastitic goat milk samples [52,53,54,55]. Several researchers also found high levels of coagulase-negative Staphylococci (CNS), which often inhabit the skin of mammary glands in small ruminants and may also express some resistance to antibiotics [25,56]. The environmental streptococci strains have been linked to poorer hygiene practices and inappropriate bedding of goats [57]. This finding has also been highlighted in the cow sector, where streptococcal intramammary infections show a high prevalence in cows being milked by hand [58]. Also, the progress of the lactation stage affected the prevalence of *Streptococcus* spp. (odds ratio 2.7), possibly due to an increased sensitivity of the udder to intramammary infections as lactation proceeded [59].

It is noteworthy that 20% of samples tested negative in the extensive farms and 25% tested negative in the intensive goat farms. It is important to consider the apocrine type of the mammary gland in goats and its distinct function during various physiology stages, which is sometimes responsible for rising somatic cell counts in milk [60,61,62] without any infection occurring. These results could justify this function; a non-infectious cause that leads to elevated SCC.

## 4. Conclusions

This study assessed the concentration of aflatoxin M1, its seasonal variation, and the bacterial profile (infectious agents responsible for mastitis) of raw goat milk of Skopelos goats under two different farming systems with different management practices. In conclusion, the farming system affects the content of aflatoxin M1 in produced milk and the prevalence of bacterial infections that may induce mastitis in dairy goats.

Intensive conditions in dairy goat farms, where concentrates are used daily, increase the possibility of elevated levels of AFM1 in goat milk. This finding confirms the impact of production methods on the aflatoxin contamination of goat milk. In addition, cold weather along with high humidity levels can also affect the AFM1 concentration in goat milk, although this was not demonstrated in the present study. A significant interaction effect was observed between year, season, and farming system on the concentration of AFM1 in goat milk. Moreover, in this study, the examination of milk samples from animals suspected of subclinical mastitis revealed that goats from the extensive system were prone to streptococcal infections, while the prevalence of *Streptococcus* spp. varied significantly between the farming systems. Also, the susceptibility of animals to mastitis-causing bacteria seemed to increase as lactation progressed.

## Figures and Tables

**Table 1:** Distribution of goat milk samples by AFM1 (ng/kg) per farming system.

Farming System	AFM1 Levels (ng/kg)			
	<5	<25	25–50	>50
Extensive	81	31	-	-
Intensive	62	54	3	2

**Table 2:** Means of AFM1 and AFB1 content from different farming systems, seasons, and years.

	FS		S		Y		*p*-Value			
Aflatoxins	Extensive	Intensive	Winter	Summer	2022	2023	FS	S	Y	FS × S × Y
AFM1 (ng/kg)	3.78 ± 0.79	7.76 ± 0.76	6.42 ± 1.10	5.12 ± 1.10	6.48 ± 0.76	5.04 ± 0.76	<0.01	0.21	0.20	0.004

AFM1: aflatoxin M1; means ± standard error (S.E). FS; farming system; S: season; Y: year; significance is observed for *p*-value < 0.05.

**Table 3:** Coefficients, standard errors, *p*-values, and odds ratio of binomial regression for the effect of farming system.

Variable	B	S.E	Sig	Exp(B)
Farming system	0.65	0.31	0.03	1.9
Lactation stage	0.98	0.35	0.005	2.7

B: regression coefficient. S.E: standard error. Sig: significance. Exp(B): odds ratio

## Data Availability

The original contributions presented in the study are included in the article; further inquiries can be directed to the corresponding author.

## Abbreviations

The following abbreviations are used in this manuscript:

AFM1	Aflatoxin M1
AFB1	Aflatoxin B1
SCC	Somatic Cell Count
EFSA	European Food and Safety Authority
FDA	Food and Drug Administration
TBC	Total Bacterial Count
CNS	Coagulase-Negative Staphylococci
ELISA	Enzyme-Linked Immunosorbent Assay
CNA	Columbia Naladixic Acid

## References

[1] Guevara-Gonzalez R.G. (2011). Aflatoxins—Biochemistry and Molecular Biology.

[2] Ismail A., Akhtar S., Levin R.E., Ismail T., Riaz M., Amir M. (2016). Aflatoxin M1: Prevalence and Decontamination Strategies in Milk and Milk Products. Crit. Rev. Microbiol..

[3] Seid A., Mama A. (2019). Aflatoxicosis and Occurrence of Aflatoxin M1 (AFM1) in Milk and Dairy Products: A Review. Austin J. Vet. Sci. Anim. Husb..

[4] Nguyen T., Flint S., Palmer J. (2020). Control of Aflatoxin M1 in Milk by Novel Methods: A review. Food Chem..

[5] Vaz A., Cabral Silva A.C., Rodrigues P., Venâncio A. (2020). Detection Methods for Aflatoxin M1 in Dairy Products. Microorganisms.

[6] Kolarič L., Minarovičová L., Lauková M., Kohajdová Z., Šimko P. (2024). Elimination of Aflatoxin M1 from Milk: Current Status, and Potential Outline of Applicable Mitigation Procedures. Trends Food Sci. Technol..

[7] Setsetse K.G. (2019). The Impact of Storage Facilities on Animal Feed Quality with Reference to Mycotoxin Contamination Around Ngaka Modiri Molema District, North West Province. Ph.D. Thesis.

[8] Rainard P. (2017). Mammary Microbiota of Dairy Ruminants: Fact or Fiction?. Vet. Res..

[9] Neculai-Valeanu A.-S., Ariton A.-M. (2022). Udder Health Monitoring for Prevention of Bovine Mastitis and Improvement of Milk Quality. Bioengineering.

[10] European Commission (2004). Regulation (EC) No 853/2004 of the European Parliament and of the Council of 29 April 2004 Laying down Specific Hygiene Rules for Food of Animal Origin. Off. J. L.

[11] European Commission (2005). Commission Regulation (EC) No 2073/2005 of 15 November 2005 on Microbiological Criteria for Foodstuffs. Off. J. Eur. Union.

[12] van Egmond H.P. (1989). Mycotoxins in Dairy Products.

[13] Kos J., Lević J., Đuragić O., Kokić B., Miladinović I. (2014). Occurrence and Estimation of Aflatoxin M1 Exposure in Milk in Serbia. Food Control.

[14] Iqbal S.Z., Jinap S., Pirouz A.A., Faizal A.R.A. (2015). Aflatoxin M1 in Milk and Dairy Products, Occurrence and Recent Challenges: A Review. Trends Food Sci. Technol..

[15] Silva I.M.D.M., Da Cruz A.G., Ali S., Freire L.G.D., Fonseca L.M., Rosim R.E., Corassin C.H., Oliveira R.B.A.D., Oliveira C.A.F.D. (2023). Incidence and Levels of Aflatoxin M1 in Artisanal and Manufactured Cheese in Pernambuco State, Brazil. Toxins.

[16] Pietri A., Mulazzi A., Piva G., Bertuzzi T. (2016). Fate of Aflatoxin M 1 During Production and Storage of Parmesan Cheese. Food Control.

[17] Elkak A., El Atat O., Habib J., Abbas M. (2012). Occurrence of Aflatoxin M1 in Cheese Processed and Marketed in Lebanon. Food Control.

[18] Motawee M.M., McMahon D.J. (2009). Fate of Aflatoxin M1 During Manufacture and Storage of Feta Cheese. J. Food Sci..

[19] European Commission (2023). Commission Regulation (EU) 2023/915 of 25 April 2023 on Maximum Levels for Certain Contaminants in Food and Repealing Regulation (EC) No 1881/2006. Off. J. Eur. Union.

[20] FDA (2005). Drug Administration. Compliance Policy Guide (CPG) Sec 527.400 Whole Milk, Lowfat Milk, Skim Milk-Aflatoxin M1.

[21] Gonzales-Barron U., Gonçalves-Tenório A., Rodrigues V., Cadavez V. (2017). Foodborne Pathogens in Raw Milk and Cheese of Sheep and Goat Origin: A Meta-Analysis Approach. Curr. Opin. Food Sci..

[22] Maréchal L., Loir L., Maréchal C.L., Thiéry R., Vautor E., Loir Y.L., Maréchal C.L., Loir Y.L., Thiéry R. (2011). Mastitis Impact on Technological Properties of Milk and Quality of Milk Products—A Review. Dairy Sci. Technol..

[23] Auldist M.J., Hubble I.B. (1998). Effects of Mastitis on Raw Milk and Dairy Products. Aust. J. Dairy Technol..

[24] Akers R.M., Nickerson S.C. (2011). Mastitis and Its Impact on Structure and Function in the Ruminant Mammary Gland. J. Mammary Gland Biol. Neoplasia.

[25] Nelli A., Voidarou C., Venardou B., Fotou K., Tsinas A., Bonos E., Fthenakis G.C., Skoufos I., Tzora A. (2022). Antimicrobial and Methicillin Resistance Pattern of Potential Mastitis-Inducing *Staphylococcus aureus* and Coagulase-Negative Staphylococci Isolates from the Mammary Secretion of Dairy Goats. Biology.

[26] Bhagya J.N., Tresamol P.V., Vijayakumar K., Shyma V.H., Mini M. (2022). Molecular Detection of Methicillin Resistant *Staphylococcus aureus* Associated with Mastitis in Goats. J. Vet. Anim. Sci..

[27] Rahimi E., Sepehri S., Safarpoor Dehkordi F., Shaygan S., Momtaz H. (2014). Prevalence of Yersinia Species in Traditional and Commercial Dairy Products in Isfahan Province, Iran. Jundishapur J. Microbiol..

[28] Obaidat M.M., Salman A.E.B., Roess A.A. (2018). High Prevalence and Antimicrobial Resistance of mecA *Staphylococcus aureus* in Dairy Cattle, Sheep, and Goat Bulk Tank Milk in Jordan. Trop. Anim. Health Prod..

[29] Cavicchioli V.Q., Scatamburlo T.M., Yamazi A.K., Pieri F.A., Nero L.A. (2015). Occurrence of *Salmonella*, *Listeria monocytogenes*, and Enterotoxigenic *Staphylococcus* in Goat Milk from Small and Medium-Sized Farms Located in Minas Gerais State, Brazil. J. Dairy Sci..

[30] Xing X., Zhang Y., Wu Q., Wang X., Ge W., Wu C. (2016). Prevalence and Characterization of *Staphylococcus aureus* Isolated from Goat Milk Powder Processing Plants. Food Control.

[31] Yaniarti M.N., Amarantini C., Budiarso T.Y. (2017). The Effect of Temperature and Pasteurization Time on *Staphylococcus aureus* Isolates from Dairy Products. AIP Conference Proceedings.

[32] Danmallam F.A., Pimenov N.V. (2019). Study on Prevalence, Clinical Presentation, and Associated Bacterial Pathogens of Goat Mastitis in Bauchi, Plateau, and Edo States, Nigeria. Vet. World.

[33] Rovai M., Caja G., Salama A.A.K., Jubert A., Lázaro B., Lázaro M., Leitner G. (2014). Identifying the Major Bacteria Causing Intramammary Infections in Individual Milk Samples of Sheep and Goats Using Traditional Bacteria Culturing and Real-Time Polymerase Chain Reaction. J. Dairy Sci..

[34] Tzora A., Skoufos J., Tsinas A., Fotou K., Karamoutsios A., Kalyva Z., Nikolaou K., Fthenakis G.C. (2016). The Bacterial Flora of the Udder of Goats. J. Hell. Vet. Med. Soc..

[35] Abdelrahman M.A., Khadr A.M., Mahmoud A.A., Elsheimy T.M., Osman A. (2020). Occurrence of Clinical and Subclinical Mastitis and Associated Risk Factors in Lactating Goats with Special Reference to Dry Period Infection and Teat Skin. Alex. J. Vet. Sci..

[36] Tenhagen B.-A., Hansen I., Reinecke A., Heuwieser W. (2009). Prevalence of Pathogens in Milk Samples of Dairy Cows with Clinical Mastitis and in Heifers at First Parturition. J. Dairy Res..

[37] Virdis S., Corgiolu G., Scarano C., Pilo A.L., De Santis E.P.L. (2008). Occurrence of Aflatoxin M1 in Tank Bulk Goat Milk and Ripened Goat Cheese. Food Control.

[38] Waqas M., Pervaiz W., Zia K.M., Iqbal S.Z. (2021). Assessment of Aflatoxin B1 in Animal Feed and Aflatoxin M1 in Raw Milk Samples of Different Species of Milking Animals from Punjab, Pakistan. Wiley Online Libr..

[39] Bingöl N.T., Dede S., Yuzuncu V., Ceylan E. (2007). Influence of Aflatoxin Present in Forages and Concentrated Feding Stuffs on Milk and Some Serum Biochemical Parameters in Goats. Bull.-Vet. Inst. Pulawy.

[40] de Matos C.J., Schabo D.C., do Nascimento Y.M., Tavares J.F., de Lima E.O., da Cruz P.O., de Souza E.L., Magnani M., Magalhães H.I. (2021). Aflatoxin M 1 in Brazilian Goat Milk and Health Risk Assessment. J. Environ. Sci. Health.

[41] Fallah A.A., Fazlollahi R., Emami A. (2016). Seasonal Study of Aflatoxin M1 Contamination in Milk of Four Dairy Species in Yazd, Iran. Food Control.

[42] Asi M.R., Iqbal S.Z., Ariño A., Hussain A. (2012). Effect of Seasonal Variations and Lactation Times on Aflatoxin M1 Contamination in Milk of Different Species from Punjab, Pakistan. Food Control.

[43] Manouras A., Malissiova E. (2018). Occurrence of Aflatoxins in compound feeds and feed materials for dairy livestock in Central Greece. J. Hell. Vet. Med. Soc..

[44] Chaisri W., Mongkon W., Sugita-Konishi Y., van Dam D., Huntley I., Suriyasathaporn W. (2017). Feed and Feed Storage Factors in Relation to Aflatoxin M1 Contamination in Bulk Milk of Smallholder Dairy Farms. Mycotoxins.

[45] Makau C.M., Matofari J.W., Muliro P.S., Bebe B.O. (2016). Aflatoxin B1 and Deoxynivalenol Contamination of Dairy Feeds and Presence of Aflatoxin M1 Contamination in Milk from Smallholder Dairy Systems in Nakuru, Kenya. Int. J. Food Contam..

[46] Anyango G., Mutua F., Kagera I., AndangO P., Grace D., Lindahl J.F. (2018). A Survey of Aflatoxin M1 Contamination in Raw Milk Produced in Urban and Peri-Urban Areas of Kisumu County, Kenya. Infect. Ecol. Epidemiol..

[47] De Roma A., Rossini C., Ritieni A., Gallo P., Esposito M. (2017). A Survey on the Aflatoxin M1 Occurrence and Seasonal Variation in Buffalo and Cow Milk from Southern Italy. Food Control.

[48] Ismaiel A.A., Tharwat N.A., Sayed M.A., Gameh S.A. (2020). Two-Year Survey on the Seasonal Incidence of Aflatoxin M1 in Traditional Dairy Products in Egypt. J. Food Sci. Technol..

[49] Marogna G., Pilo C., Vidili A., Tola S., Schianchi G., Leori S.G. (2012). Comparison of Clinical Findings, Microbiological Results, and Farming Parameters in Goat Herds Affected by Recurrent Infectious Mastitis. Small Rumin. Res..

[50] Gabli Z., Djerrou Z., Gabli A.E., Bensalem M. (2019). Prevalence of Mastitis in Dairy Goat Farms in Eastern Algeria. Vet. World.

[51] Begum M., Hossain M., Ershaduzzaman M., Alam M. (2016). Epidemiological Studies on Subclinical Mastitis in Dairy Goats in Northern Regions of Bangladesh. Bangladesh J. Livest. Res..

[52] Zhao Y., Liu H., Zhao X., Gao Y., Zhang M., Chen D. (2015). Prevalence and Pathogens of Subclinical Mastitis in Dairy Goats in China. Trop. Anim. Health Prod..

[53] Ariffin M.F.T., Hasmadi N., Hian C.M., Ghazali M.F., Suhaili Z., Ariffin S.M.Z. (2020). Prevalence and Antimicrobial Sensitivity Pattern of *Staphylococcus aureus* Isolated from Clinical and Subclinical Mastitis in Small Ruminant in Besut and Setiu, Terengganu, Malaysia. Malays. J. Microbiol..

[54] Jabbar A., Saleem M.H., Iqbal M.Z., Qasim M., Ashraf M., Tolba M.M., Nasser H.A., Sajjad H., Hassan A., Imran M. (2020). Epidemiology and Antibiogram of Common Mastitis-Causing Bacteria in *Beetal goats*. Vet. World.

[55] Widianingrum D.C., Silaban D.G., Fanata W.I.D., Khasanah H. (2024). Identification of Antibiotic Resistance Genes in *Escherichia coli* from Subclinical Mastitis Milk in Dairy Cows and Goats, East Java Province. Vet. Med..

[56] Moura G.S., Gebreyes W.A., Marques M.F.S., Stipp D.T., Souza F.N., Da Costa L.B., Oliveira C.J.B. (2018). Short Communication: Occurrence of Methicillin-Resistant *Staphylococcus aureus* and Coagulase-Negative Staphylococci in Dairy Goat Herds in Ohio, United States. J. Dairy Sci..

[57] Haggag Y., Nossair M., Naggar A., Abdallah M., Habib H., Farag H. (2019). Microbiological Studies on Streptococcus Species Associated with Environmental Mastitis in Sheep and Goats Farms. Alex. J. Vet. Sci..

[58] Mathewos M., Fesseha H., Tindashe M.Y., Markos A., Yirgalem M. (2020). Study on Bovine Mastitis with Isolation, Identification and An-timicrobial Resistance Patterns of Streptococci Species from Raw Milk in Bishoftu Town, Ethiopia. SM Trop. Med. J..

[59] Albenzio M., Santillo A., Caroprese M., Ciliberti M.G., Marino R., Sevi A. (2016). Effect of Stage of Lactation on the Immune Competence of Goat Mammary Gland. J. Dairy Sci..

[60] Souza F.N., Blagitz M.G., Penna C.F.A.M., Della Libera A.M.M.P., Heinemann M.B., Cerqueira M.M.O.P. (2012). Somatic Cell Count in Small Ruminants: Friend or Foe?. Small Rumin. Res..

[61] Boyazoglu J., Morand-Fehr P. (2001). Mediterranean Dairy Sheep and Goat Products and Their Quality: A Critical Review. Small Rumin. Res..

[62] Desidera F., Skeie S.B., Devold T.G., Inglingstad R.A., Porcellato D. (2025). Fluctuations in Somatic Cell Count and Their Impact on Individual Goat Milk Quality Throughout Lactation. J. Dairy Sci..

